# Evaluation of university scientific research ability based on the output of sci-tech papers: A D-AHP approach

**DOI:** 10.1371/journal.pone.0171437

**Published:** 2017-02-17

**Authors:** Fan Zong, Lifang Wang

**Affiliations:** 1 Division of Human Resources, Northwestern Polytechnical University, Xi’an, Shaanxi 710072, P.R.China; 2 School of Management, Northwestern Polytechnical University, Xi’an, Shaanxi 710072, P.R.China; Southwest University, CHINA

## Abstract

University scientific research ability is an important indicator to express the strength of universities. In this paper, the evaluation of university scientific research ability is investigated based on the output of sci-tech papers. Four university alliances from North America, UK, Australia, and China, are selected as the case study of the university scientific research evaluation. Data coming from Thomson Reuters InCites are collected to support the evaluation. The work has contributed new framework to the issue of university scientific research ability evaluation. At first, we have established a hierarchical structure to show the factors that impact the evaluation of university scientific research ability. Then, a new MCDM method called D-AHP model is used to implement the evaluation and ranking of different university alliances, in which a data-driven approach is proposed to automatically generate the D numbers preference relations. Next, a sensitivity analysis has been given to show the impact of weights of factors and sub-factors on the evaluation result. At last, the results obtained by using different methods are compared and discussed to verify the effectiveness and reasonability of this study, and some suggestions are given to promote China’s scientific research ability.

## 1 Introduction

Research and Development (R & D) ability is a crucial indicator to reflect the innovation capability of a country. Universities, as the highest-level academic institutions, are the most important sources to produce new knowledge and accelerate the advance of human civilization. In order to better promote the development of universities and quantify the performance of universities, the evaluation of university scientific research ability is of great significance [1–5].

Recent years, some organizations regularly release university rankings, such as QS World University Rankings, US News Top World University Rankings, Times Higher Education World University Rankings, Academic Ranking of World Universities (ARWU), etc. Within these rankings, the technology of Multi-Criteria Decision Making (MCDM) [6–8] is one of the most popular methodology to implement the ranking and evaluation of world universities. Some classical MCDM methods include Analytic Hierarchy and Network Processes (AHP/ANP) [9], Technique for Order of Preference by Similarity to Ideal Solution (TOPSIS) [10], VIKOR method [11], Preference ranking organization method for enrichment evaluation (PROMETHEE) [12], Data envelopment analysis (DEA) [13], and so on.

As the rapid elevation of China’s economic strength and international status, the government invests more and more effort to promote the research performance of China’s universities. A series of ambitious programs, for example 211 Project, 985 Project, Double First-Class Project, have been carried out. And many universities have formed alliances to share educational resources and promote cooperation, so as to fast boost their scientific research ability as a whole. By investing so much resources on universities, the impact of these projects is widely concerned and the evaluation of research performance of universities has been an important research field [14, 15]. Zhang et al.’s [16] have assessed the impact of the 985 Project on increasing the rate of publication in international journals at 24 universities by using the regression analysis approach. Different from measuring research performance by simply using the Science Citation Index (SCI) at the early stage [17], Li et al. [18] presented a two-dimensional approach by balancing “quantity” and “quality” to evaluate the research performance of universities in Mainland China, Hong Kong and Taiwan. In [19], the authors have developed a framework of performance measure indicators for universities which includes 18 measurement dimensions and 78 performance measure indicators. Chen and Kenney [20] have given a comparative research on the role of universities and research institutes in development of the Beijing and Shenzhen technology clusters. Moreover, a Chinese perspective on world university ranking, Academic Ranking of World Universities [21], has been released annually since 2003, which partially provides the evaluation of Chinese universities’ performance compared with other universities around the world.

In this paper, inspired by the idea of MCDM, the evaluation of university scientific research ability has studied. Four famous university alliances including Association of American Universities (AAU) of North America, Russell group (Rg) of UK, Group of Eight (Go8) of Australia, and C9 League (C9) of China, are considered. At first, the data are collected from a well-known science information dataset—Thomson Reuters InCites [22]. Then, a hierarchical structure for the scientific research ability evaluation has been established. The proposed hierarchical structure contains three main aspects including quantity of publications, quality of publications, and influence of papers and subjects. Especially, the quantity refers to the number of Total Publications (TP), the quality includes three sub-factors which are Total Citations (TC), Citation Impact (CI), and % Documents Cited (%DC), and the influence is composed by Impact Relative to World (IRW) and Number of Preponderant Discipline (NPD). After that, a D-AHP approach [23], which is a new AHP method extended by D numbers [24], is applied to implement the evaluation and rank the four university alliances in terms of their sci-tech papers output. Within the evaluation process, a data-driven approach is proposed to automatically generate the D numbers preference relations which is also called D matrix. Next, a sensitivity analysis is presented to show the impact of weights of factors and sub-factors on the evaluation result. At last, the results obtained by using different methods are compared and discussed to verify the effectiveness and reasonability of this study, and some suggestions are given to promote China’s scientific research ability.

The remainder of this paper is organized as follows. A brief review about China’s key programs on improving universities’ scientific research ability is given in section 2. A brief introduction about methodology including D numbers and D-AHP approach is presented in section 3. Then, the evaluation objects and data are collected in section 4. After that, the evaluation process of university scientific research ability using the D-AHP approach is illustrated is section 5. Next, a sensitivity analysis is given in section 6. Comparison and discussion among different methods on the study are shown in section 7. Finally, section 8 concludes the paper.

## 2 Review of China’s key programs on improving universities’ scientific research ability

With the fast progress of China’s economic strength, as the intellectual foundation and talent reserve for sustainable development, higher eduction has been placed on more and more important status by Chinese government. The governments, either the central or local, have implemented a series of programs to improve the scientific research ability of China’s universities. Some of the most important programs are reviewed as follows.

From 1995, the Chinese central government has implement a project entitled “High-level Universities and Key Disciplinary Fields”, as known as 211 Project, to create around 100 world class universities as a national priority for the 21st century to meet the demands of socio-economic development. Now there are 112 universities designated as 211 Project institutions which could receive focused support from the government including funding, construction of key laboratories, student enrollment right, and so on. From 1996 to 2000, during the first phase of the project, approximately 2.2 billion US dollars was distributed among the 211 Project universities [25]. The impact of the project to the participating universities is enormous, a typical case is given in [26] which takes Yanbian university as an example.

In 1998, a project named as 985 Project was announced by Chinese President Jiang Zemin at the Centenary Celebration of Beijing University. The 985 Project is entitled “World Class Universities” which is exactly consistent with its goal that is to build a number of first-rate universities of international advanced level. Currently, there are 39 universities participating in the 985 Project. Zhang et al. [16] have presented a work to assess the impact of the 985 Project. According to their research, after the implementation of the 985 Project the growth rate of publications for the 985 Project universities increases more quickly. Additionally, the discussion and reflection on the effects of the 985 Project have also been concerned [27, 28].

The 211 Project and 985 Project are the two most important projects for improving the research performance of China’s universities, currently both of them are prohibited to the participation of new universities. As the progress and continuation of 211 Project and 985 Project, the Higher Education Innovative Capacity Improvement Project or 2011 Project was developed in light of Chinese President Hu Jintao’s speech at Tsinghua University in 2011. This project aims to improve the innovation capability of universities and research institutions through a mechanism of collaborative partnerships, so as to speed up the establishment of China as an innovative country generating high quality and relevant research outcomes. In addition to these projects mentioned above, the central government of China has successively worked out a series of other projects for revitalizing China’s higher education and research & development strength, for examples 111 Project which aims to attract high-level talents to build a number of world class innovation bases, and 985 Project Innovation Platform that endeavors in constructing high-level innovation platforms for some designated key disciplines, and National Basic Ability Construction Project of Western and Central China that is for the revitalization of higher education in western and central China. Now a new major plan is implementing, which is called “Double First-Class Project” unofficially that is an upgraded version of the former 985 Project and 211 Project, and it is designed to construct a number of world-class universities and disciplines by 2020 and 2030.

With the leap of China’s higher education strength, a number of university alliances, analogous to the AAU in the US, the Go8 in Australia, and Russell group in the UK, have been formed officially or unofficially. The top 1 university alliance in China is called C9 League which consists of 9 elite universities. C9 League is the Chinese version of Ivy League. In addition, other famous university alliances in China include the Excellence League composed by 10 excellent technological universities, University Alliance of the New Silk Road (UANSR), E8 which consists of 8 key universities located in the delta region of Yangtze river, Federation of Beijing Hi-Tech Universities (12 schools located in Beijing ares), Z14 which is composed by 14 universities from western and central China, etc. By considering the vast investment, how to scientifically evaluate the university scientific research ability of different university alliances has been an important issue which is our concern in this study.

## 3 Methodology

### 3.1 D numbers

D numbers [23, 24, 29, 30] is a new model of representing and handling uncertain information, which is an effective extension of the basic probability assignment (BPA) of Dempster-Shafer evidence theory [31–36]. Theoretically, D numbers overcomes two typical deficiencies of Dempster-Shafer theroy, namely exclusiveness hypothesis and completeness constraint. Since its advantages in dealing with uncertain information, D numbers has attracted increasing attention and been used in environment impact assessment [29], supplier selection [23], failure mode and effects analysis [37], new produce development [38], curtain grouting efficiency assessment [39], etc. Some basic knowledge about D numbers are given as follows.

**Definition 1** *Let* Ω *be a finite nonempty set, D numbers is a mapping formulated by*

D:Ω→[0,1] (1)

*with*

$$\begin{array}{c} \sum_{B\subseteq \Omega }D(B)\leq 1\;and\;D\left(\emptyset \right)=0 \end{array}$$ (2)

*where* ∅ *is an empty set and B is a subset of* Ω.

If $\sum_{B\subseteq \Omega }D(B)=1$, the information is complete; If $\sum_{B\subseteq \Omega }D(B)<1$, the information is incomplete. An illustrative example is given to show a D numbers as below.

**Example 1** *Suppose a project is assessed, the assessment score is represented by interval* [0, 100]. *In the frame of D numbers, an expert may give the assessment as follows:*

$$\begin{array}{c} \begin{array}{c} D(\left\{b_{1}\right\})=0.4\\ D(\left\{b_{3}\right\})=0.1\\ D(\left\{b_{1},b_{2},b_{3}\right\})=0.4 \end{array} \end{array}$$

*where* *b*_1_ = [0, 20], *b*_2_ = [35, 65], *b*_3_ = [40, 100]. *Here, since* *D*({*b*_1_}) + *D*({*b*_3_}) + *D*({*b*_1_, *b*_2_, *b*_3_}) = 0.9, *it indicates that the information is incomplete in this D numbers. What’s more important, the elements in the set of* {*b*_1_, *b*_2_, *b*_3_} *are not mutually exclusive in the D numbers*.

For a discrete set Ω = {*b*_1_, *b*_2_, ⋯, *b*_*i*_, ⋯, *b*_*n*_}, where *b*_*i*_ ∈ *R* and *b*_*i*_ ≠ *b*_*j*_ if *i* ≠ *j*, a special form of D numbers can be expressed by

$$\begin{array}{cc} D\left(\left\{b_{1}\right\}\right) & =v_{1}\\ D\left(\left\{b_{2}\right\}\right) & =v_{2}\\ \cdots & \cdots \\ D\left(\left\{b_{i}\right\}\right) & =v_{i}\\ \cdots & \cdots \\ D\left(\left\{b_{n}\right\}\right) & =v_{n} \end{array}$$ (3)

or simply denoted as *D* = {(*b*_1_, *v*_1_), (*b*_2_, *v*_2_), ⋯, (*b*_*i*_, *v*_*i*_), ⋯, (*b*_*n*_, *v*_*n*_)}, or $D=(\begin{array}{cc} b_{1} & v_{1}\\ b_{2} & v_{2}\\ \vdots & \vdots \\ b_{n} & v_{n} \end{array})$, where *v*_*i*_ > 0 and $\sum_{i=1}^{n}v_{i}\leq 1$.

D numbers has the following properties which come from literature [29].

**Definition 2** *Permutation invariability. If there are two D numbers*

$$D_{1}=\left\{\left(b_{1},v_{1}\right),\cdots ,\left(b_{i},v_{i}\right),\cdots ,\left(b_{n},v_{n}\right)\right\}$$

*and*

$$D_{2}=\left\{\left(b_{n},v_{n}\right),\cdots ,\left(b_{i},v_{i}\right),\cdots ,\left(b_{1},v_{1}\right)\right\},$$

*then* *D*_1_ ⇔ *D*_2_.

**Example 2** *If there are two D numbers:*

$$D_{1}=\left\{\left(0,0.7\right),\left(1,0.3\right)\right\}\;and\;D_{2}=\left\{\left(1,0.3\right),\left(0,0.7\right)\right\}$$

*Then*

$$D_{1}\Leftrightarrow D_{2}$$

**Definition 3** *For* *D* = {(*b*_1_, *v*_1_), (*b*_2_, *v*_2_), ⋯, (*b*_*i*_, *v*_*i*_), ⋯, (*b*_*n*_, *v*_*n*_)}, *the integration representation of D is defined as*

$$I\left(D\right)=\sum_{i=1}^{n}b_{i}v_{i}$$ (4)

*where* *b*_*i*_ ∈ *R*, *v*_*i*_ > 0 *and* $\sum_{i=1}^{n}v_{i}\leq 1$.

**Example 3** *Let* *D* = {(1, 0.2), (2, 0.1), (3, 0.3), (4, 0.3), (5, 0.1)}, *Then*

I(D)=1×0.2+2×0.1+3×0.3+4×0.3+5×0.1=3.0

In addition, in References [24, 29, 38], the authors addressed the combination rules of D numbers, and the distance function of D numbers. These studies have further enriched the theoretical framework of D numbers.

### 3.2 D-AHP approach

**Fig 1:**
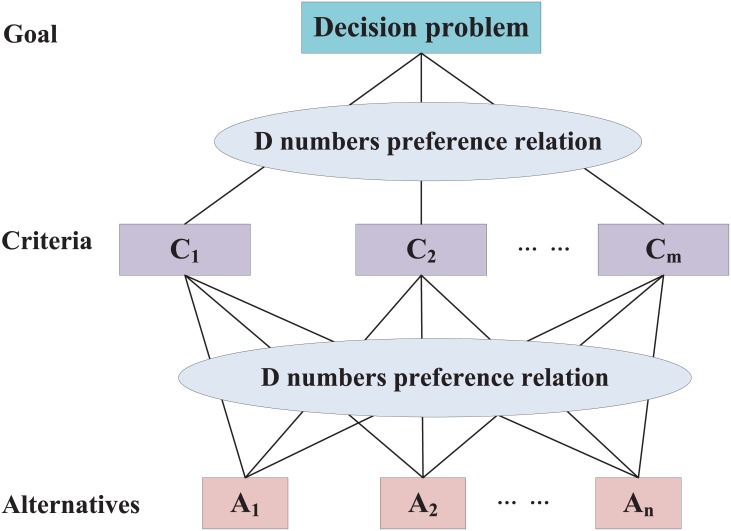
The framework of D-AHP approach [23].

The D-AHP approach was first proposed in literature [23] to solve the supplier selection problem under uncertain environment. As the first model based on D numbers, the D-AHP approach has extend the classical AHP method, as shown in Fig 1. Similar to the AHP method, the D-AHP model also has three levels, including goal, criteria, and alternatives. Ant it still uses the weighted averaging method to integrate the weights in each levels, as shown in Table 1. However, within the D-AHP model the pairwise comparison matrix is replaced by the D numbers preference relation which is also called as D matrix.

**Table 1:** The integration of each level’s weights in D-AHP [23].

	*C*_1_ *c*_1_	*C*_2_ *c*_2_	⋯ ⋯	*C*_*m*_ *c*_*m*_	Alternatives’ weights for the decision problem
*A*_1_	*a*_11_	*a*_12_	…	*a*_1*m*_	$w_{1}=\sum_{i=1}^{m}c_{i}a_{1i}$
*A*_2_	*a*_21_	*a*_22_	…	*a*_2*m*_	$w_{2}=\sum_{i=1}^{m}c_{i}a_{2i}$
⋮	⋮	⋮	⋱	⋮	⋮
*A*_*n*_	*a*_*n*1_	*a*_*n*2_	…	*a*_*nm*_	$w_{n}=\sum_{i=1}^{m}c_{i}a_{ni}$

Essentially, D matrix is a fuzzy preference relation extended by D numbers. The conventional fuzzy preference relation [40–42] is represented by a *n* × *n* matrix *R* = [*r*_*ij*_]_*n*×*n*_ having the following form:

$$R=\begin{array}{cc} & \begin{array}{cccc} A_{1} & A_{2} & \cdots & A_{n} \end{array}\\ \begin{array}{c} A_{1}\\ A_{2}\\ \vdots \\ A_{n} \end{array} & \left[\begin{array}{cccc} r_{11} & r_{12} & \cdots & r_{1n}\\ r_{21} & r_{22} & \cdots & r_{2n}\\ \vdots & \vdots & \ddots & \vdots \\ r_{n1} & r_{n2} & \cdots & r_{nn} \end{array}\right] \end{array}$$ (5)

where (i) *r*_*ij*_ ≥ 0; (ii) *r*_*ij*_ + *r*_*ji*_ = 1, ∀*i*, *j* ∈ {1, 2, ⋯, *n*}; (iii) *r*_*ii*_ = 0.5, ∀*i* ∈ {1, 2, ⋯, *n*}. And *r*_*ij*_ = *μ*_*R*_(*A*_*i*_, *A*_*j*_) denotes the preference degree of alternative *A*_*i*_ over alternative *A*_*j*_. Here, *r*_*ij*_ = 0 means *A*_*j*_ is absolutely preferred to *A*_*i*_; *r*_*ij*_ < 0.5 means *A*_*j*_ is preferred to *A*_*i*_ to some degree; *r*_*ij*_ = 0.5 means indifference between *A*_*i*_ and *A*_*j*_; *r*_*ij*_ > 0.5 means *A*_*i*_ is preferred to *A*_*j*_ to some degree; *r*_*ij*_ = 1 means *A*_*i*_ is absolutely preferred to *A*_*j*_. By contrast, a D matrix is

$$R_{D}=\begin{array}{cc} & \begin{array}{cccc} A_{1}\; & A_{2}\; & \cdots & A_{n} \end{array}\\ \begin{array}{c} A_{1}\\ A_{2}\\ \vdots \\ A_{n} \end{array} & \left[\begin{array}{cccc} D_{11} & D_{12} & \cdots & D_{1n}\\ D_{21} & D_{22} & \cdots & D_{2n}\\ \vdots & \vdots & \ddots & \vdots \\ D_{n1} & D_{n2} & \cdots & D_{nn} \end{array}\right] \end{array}$$ (6)

where $D_{ij}=\left\{\left(b_{ij}^{1},v_{ij}^{1}\right),\left(b_{ij}^{2},v_{ij}^{2}\right),\cdots ,\left(b_{ij}^{p},v_{ij}^{p}\right),\cdots \right\}$, $D_{ji}=\neg D_{ij}=\left\{\left(1-b_{ij}^{1},v_{ij}^{1}\right),\left(1-b_{ij}^{2},v_{ij}^{2}\right),\cdots ,\left(1-b_{ij}^{p},v_{ij}^{p}\right),\cdots \right\},\;\forall i,j\in \left\{1,2,\cdots ,n\right\}$, and $b_{ij}^{p}\in \left[0,1\right]$, $v_{ij}^{p}>0$, $\sum_{p}v_{ij}^{p}=1$. Obviously, *D*_*ii*_ = {(0.5, 1.0)}∀*i* ∈ {1, 2, ⋯, *n*} in *R*_*D*_.

**Fig 2:**
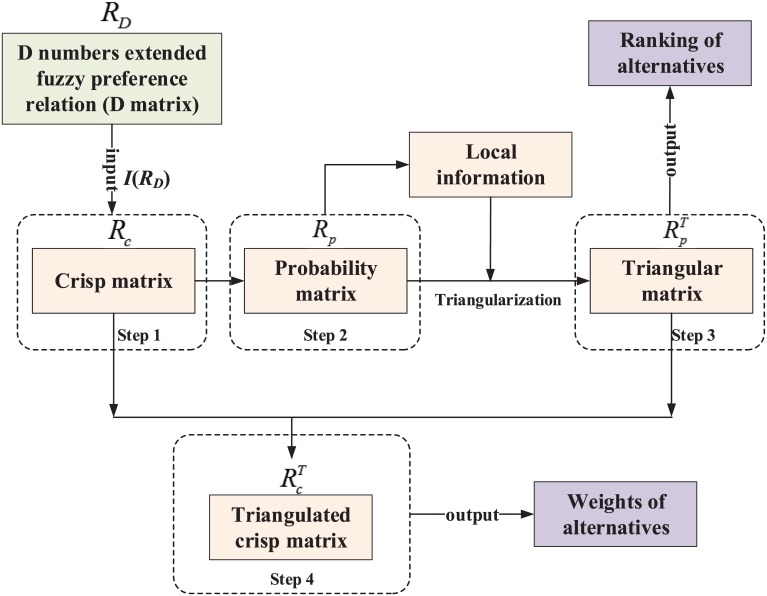
The procedure to obtain the ranking and weights of alternatives according to a D matrix [23].

A key point in the D-AHP model is how to obtain the weight of each alternative according to the D matrix. In order to solve that problem, literature [23] proposed a unified framework to obtain the ranking and weights of alternatives according to a D matrix, as shown in Fig 2. Briefly, it contains four steps.

- At first, a D matrix is seen as an input to obtain its corresponding crisp matrix *R*_*c*_ by using the integration representation of D numbers given in Eq (4).
- Second, construct a probability matrix *R*_*p*_ based on *R*_*I*_.
- Third, convert the probability matrix *R*_*p*_ to triangular matrix of probability $R_{p}^{T}$.
- At last, integrate the crisp matrix *R*_*c*_ and triangular matrix $R_{p}^{T}$ to derive triangulated crisp matrix $R_{c}^{T}$, so as to generate the weights of alternatives.

For more details about the procedure of solving a D matrix, please refer to literature [23]. In the following section, a numerical example will also be given to illustrate the calculation process in detail.

## 4 Evaluation objects and data

In this paper, four representative university alliances are selected to show the process of evaluating and comparing the scientific research ability of different universities. The four university alliances are: (1)“C9” which is an alliance of 9 prestigious Chinese universities including Peking University, Tsinghua University, Fudan University, Shanghai Jiao Tong University, Nanjing University, University of Science and Technology of China, Zhejiang University, Xi’an Jiao Tong University, Harbin Institute of Technology; (2) “Go8” which is a coalition of leading Australian universities, intensive in research and comprehensive in general and professional education, including Monash University, Australian National University, University of Adelaide, University of Melbourne, University of Queensland, University of Sydney, University of Western Australia, UNSW Australia; (3) “Rg” which represents a group of 24 leading universities in UK; and (4) “AAU” which is a nonprofit organization that comprises 62 leading public and private research universities in the United States and Canada.

With respect to the data, they are from a well-known science information dataset—Thomson Reuters InCites. In the study, we have collected the related data from 2003 to 2013. These data include three categories which are the quantity of papers, quality of papers, and influence of papers and subjects. They are introduced in the InCites Indicators Handbook [22] in detail. Here, the related indicators are given briefly as follows.

### 4.1 “Quantity”

In the paper, quantity is the amount of Total Publications (TP) within a period of time. Table 2 gives the quantity of published papers for these four university alliances from 2003 to 2013.

**Table 2:** Data of “Quantity” for the four university alliances.

University alliance	TP	Percentage to World
AAU	2,071,303	16.80
Rg	629,399	5.10
Go8	239,953	1.95
C9	297,302	2.41

### 4.2 “Quality”

The quality of papers includes three sub-factors which are Total Citations (TC), Citation Impact (CI), and % Documents Cited (%DC), respectively. Total citations is the number of total citations within a period of time. Citation impact of a set of publications is calculated by dividing the total number of citations by the total number of publications. Citation impact shows the average number of citations that a publication has received. The %DC indicator is the percentage of publications, in a set, that has received at least one citation. The data of “Quality” for the four university alliances is collected as Table 3.

**Table 3:** Data of “Quality” for the four university alliances.

University alliance	TC	CI	%DC
AAU	41,098,626	19.84	85.04
Rg	11,221,598	17.83	84.07
Go8	3,433,660	14.31	82.34
C9	2,679,909	9.01	75.14

### 4.3 “Influence”

The influence includes two aspects. One is the Impact Relative to World (IRW) which is the ratio of the Citation Impact of a set of documents divided by the world Citation Impact for a given period of time. This indicator shows the impact of the research in relation to the impact of the global research and is an indicator of relative research performance. The world average is always equal to one. If the numerical value of the Impact Relative to World exceeds one, then the assessed entity is performing above the world average. If it is less than one, then it performs below the world average. Table 4 gives the IRW for these four university alliances including AAU, Rg, Go8, and C9.

**Table 4:** The IRW of AAU, Rg, Go8, and C9.

University alliance	IRW
AAU	1.70
Rg	1.53
Go8	1.23
C9	0.77

The other one is the Number of Preponderant Discipline (NPD) which is based on the IRW in particular subject areas. Table 5 gives the IRW of each university alliance in different disciplines. For a discipline *A*, if its numerical value of the IRW is greater than one, we claim that it is a preponderant discipline belonging to a university alliance. Therefore, the NPD can be an indicator to show the research strength of an institution. From Table 5, it is found that the NPD of AAU, Rg, Go8, and C9 are 22, 22, 20, and 3, respectively.

**Table 5:** The IRW in different disciplines of AAU, Rg, Go8, and C9.

Discipline	AAU	Rg	Go8	C9
Agricultural Sciences	1.50	1.82	1.29	1.00
Biology & Biochemistry	1.47	1.33	1.10	0.62
Chemistry	1.94	1.52	1.24	0.98
Clinical Medicine	1.56	1.56	1.27	0.61
Computer Science	1.89	1.18	1.16	0.62
Economics & Business	1.85	1.13	0.81	0.71
Engineering	1.56	1.25	1.32	0.92
Environment/Ecology	1.62	1.52	1.31	0.77
Geosciences	1.65	1.55	1.27	0.90
Immunology	1.41	1.24	1.11	0.53
Mathematics	1.65	1.24	1.20	1.02
Materials Science	2.22	1.68	1.38	0.97
Microbiology	1.53	1.51	1.16	0.57
Molecular Biology & Genetics	1.48	1.37	1.04	0.50
Multidisciplinary	1.67	1.26	1.19	0.54
Neuroscience & Behavior	1.44	1.47	0.98	0.56
Pharmacology & Toxicology	1.45	1.50	1.22	0.75
Physics	1.85	1.57	1.34	0.85
Plant & Animal Science	1.59	1.79	1.39	1.09
Psychiatry/Psychology	1.46	1.34	1.04	0.56
Social Sciences, General	1.48	1.28	1.04	0.95
Space Science	1.50	1.47	1.23	0.62

### 4.4 Summarization of data

Based on the respective data as shown above, we can summarize all of data, as shown in Table 6. Now, the goal is to evaluate and compare the scientific research ability of AAU, Rg, Go8, and C9, according to Table 6.

**Table 6:** Data collection.

Factor	Sub-factor	AAU	Rg	Go8	C9
Quantity					
	TP	2,071,303	629,399	239,953	297,302
Quality					
	TC	41,098,626	11,221,598	3,433,660	2,679,909
	CI	19.84	17.83	14.31	9.01
	%DC	85.04	84.07	82.34	75.14
Influence					
	IRW	1.70	1.53	1.23	0.77
	NPD	22	22	20	3

## 5 Evaluation of university scientific research ability using the D-AHP approach

In this section, the process of using the D-AHP approach to evaluate university scientific research ability is illustrated based on the data collected in above section.

### 5.1 Hierarchical structure for the scientific research ability evaluation

**Fig 3:**
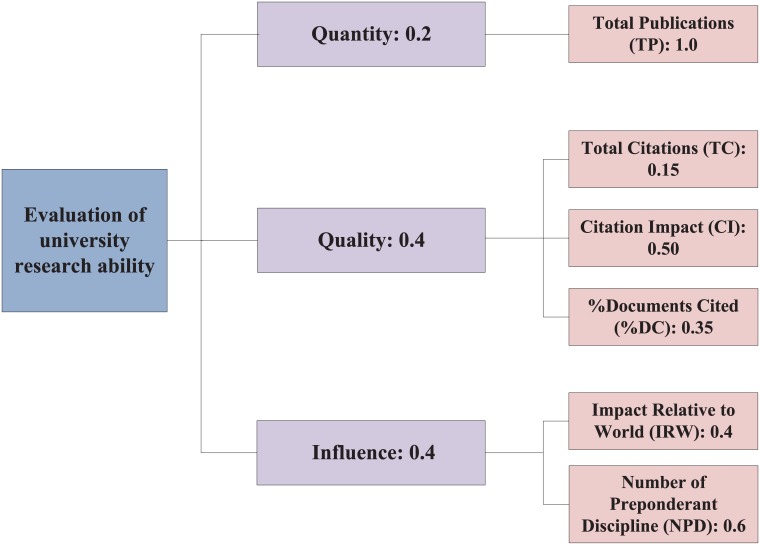
A hierarchical structure for the scientific research ability evaluation.

By consulting with the domain experts, we build a hierarchical structure for the scientific research ability evaluation which mainly determines the relative weight of each factors in different level, as shown in Fig 3. According to Fig 3, the absolute weight of each sub-factor can be calculated, as given in Table 7. From Table 7, NPD has the biggest weight for the scientific research ability evaluation, and TC is of the least weight for the evaluation. Next we can use the D-AHP approach to evaluate the scientific research ability of different university alliances.

**Table 7:** The absolute weight of each sub-factor.

Sub-factor	TP	TC	CI	%DC	IRW	NPD
Absolute weight	0.20	0.06	0.20	0.14	0.16	0.24

### 5.2 Construction of D matrix

In order to implement the scientific research ability evaluation based on the D-AHP approach, the key step is to construct the D numbers preference relation, namely D matrix. In the paper, a data-driven approach is proposed to generate the D matrix as follows.

Let us use the preference relation between AAU and Rg as the example. For AAU and Rg, according to Table 6 the TP of AAU is 2,071,303, that of Rg is 629,399. So the sum of TP of AAU and Rg is equal to 2,700,702, where AAU is with a percentage of 76.69%, and Rg is with a percentage of 23.31%. It implies that, on factor TP, AAU performs better than Rg with a preference degree of 0.7669, and Rg performs better than AAU with a preference degree of 0.2331. Therefore, *u*(*AAU*, *Rg*) = 0.7669 and *u*(*Rg*, *AAU*) = 0.2331. However, due to the absolute weight of TP is 0.20, the belief of *u*(*AAU*, *Rg*) = 0.7669 should be 0.20. Therefore, similarly, we have:

- On TC, the belief of *u*(*AAU*, *Rg*) = 0.7855 is 0.06;
- On CI, the belief of *u*(*AAU*, *Rg*) = 0.5267 is 0.20;
- On %DC, the belief of *u*(*AAU*, *Rg*) = 0.5029 is 0.14;
- On IRW, the belief of *u*(*AAU*, *Rg*) = 0.5263 is 0.16;
- On NPD, the belief of *u*(*AAU*, *Rg*) = 0.50 is 0.24.

As a result, the D numbers preference relation of denoting the preference degree of AAU over Rg is

$$D\left(AAU,Rg\right)=\left(\begin{array}{cc} 0.7669 & 0.2\\ 0.7855 & 0.06\\ 0.5267 & 0.2\\ 0.5029 & 0.14\\ 0.5263 & 0.16\\ 0.50 & 0.24 \end{array}\right)$$ (7)

By means of this way, the D numbers preference relations (D matrix) among AAU, Rg, Go8, C9, can been derived, which are given in Table 8.

**Table 8:** D numbers preference relations (D matrix) among AAU, Rg, Go8, C9.

	AAU	Rg	Go8	C9
AAU	$\left(\begin{array}{cc} 0.5 & 1 \end{array}\right)$	$\left(\begin{array}{cc} 0.7669 & 0.2\\ 0.7855 & 0.06\\ 0.5267 & 0.2\\ 0.5029 & 0.14\\ 0.5263 & 0.16\\ 0.5000 & 0.24 \end{array}\right)$	$\left(\begin{array}{cc} 0.8962 & 0.2\\ 0.9229 & 0.06\\ 0.5810 & 0.2\\ 0.5081 & 0.14\\ 0.5802 & 0.16\\ 0.5238 & 0.24 \end{array}\right)$	$\left(\begin{array}{cc} 0.8745 & 0.2\\ 0.9388 & 0.06\\ 0.6876 & 0.2\\ 0.5309 & 0.14\\ 0.6883 & 0.16\\ 0.8800 & 0.24 \end{array}\right)$
Rg	$\left(\begin{array}{cc} 0.2331 & 0.2\\ 0.2145 & 0.06\\ 0.4733 & 0.2\\ 0.4971 & 0.14\\ 0.4737 & 0.16\\ 0.5000 & 0.24 \end{array}\right)$	$\left(\begin{array}{cc} 0.5 & 1 \end{array}\right)$	$\left(\begin{array}{cc} 0.7240 & 0.2\\ 0.7657 & 0.06\\ 0.5548 & 0.2\\ 0.5052 & 0.14\\ 0.5543 & 0.16\\ 0.5238 & 0.24 \end{array}\right)$	$\left(\begin{array}{cc} 0.6792 & 0.2\\ 0.8072 & 0.06\\ 0.6642 & 0.2\\ 0.5280 & 0.14\\ 0.6652 & 0.16\\ 0.8800 & 0.24 \end{array}\right)$
Go8	$\left(\begin{array}{cc} 0.1038 & 0.2\\ 0.0771 & 0.06\\ 0.4190 & 0.2\\ 0.4919 & 0.14\\ 0.4198 & 0.16\\ 0.4762 & 0.24 \end{array}\right)$	$\left(\begin{array}{cc} 0.2760 & 0.2\\ 0.2343 & 0.06\\ 0.4452 & 0.2\\ 0.4948 & 0.14\\ 0.4457 & 0.16\\ 0.4762 & 0.24 \end{array}\right)$	$\left(\begin{array}{cc} 0.5 & 1 \end{array}\right)$	$\left(\begin{array}{cc} 0.4466 & 0.2\\ 0.5616 & 0.06\\ 0.6135 & 0.2\\ 0.5229 & 0.14\\ 0.6150 & 0.16\\ 0.8696 & 0.24 \end{array}\right)$
C9	$\left(\begin{array}{cc} 0.1255 & 0.2\\ 0.0612 & 0.06\\ 0.3124 & 0.2\\ 0.4691 & 0.14\\ 0.3117 & 0.16\\ 0.1200 & 0.24 \end{array}\right)$	$\left(\begin{array}{cc} 0.3208 & 0.2\\ 0.1928 & 0.06\\ 0.3358 & 0.2\\ 0.4720 & 0.14\\ 0.3348 & 0.16\\ 0.1200 & 0.24 \end{array}\right)$	$\left(\begin{array}{cc} 0.5534 & 0.2\\ 0.4384 & 0.06\\ 0.3865 & 0.2\\ 0.4771 & 0.14\\ 0.3850 & 0.16\\ 0.1304 & 0.24 \end{array}\right)$	$\left(\begin{array}{cc} 0.5 & 1 \end{array}\right)$

### 5.3 Solving the D matrix

Once the D matrix has been constructed, the approach shown in Fig 2 can be used to solve it so as to obtain the priority weights and ranking of university alliances. Let us present the process step by step.

At first, based on Eq (4), the D matrix shown in Table 8 is converted to a crisp matrix

$$R_{c}=\begin{array}{cc} & \begin{array}{cccc} AAU & \;Rg & \;Go8 & \;\;C9 \end{array}\\ \begin{array}{c} AAU\\ Rg\\ Go8\\ C9 \end{array} & \left(\begin{array}{cccc} 0.5000 & 0.5805 & 0.6405 & 0.7644\\ 0.4195 & 0.5000 & 0.5868 & 0.7087\\ 0.3595 & 0.4132 & 0.5000 & 0.6260\\ 0.2356 & 0.2913 & 0.3740 & 0.5000 \end{array}\right) \end{array}$$ (8)

Second, according to the crisp matrix *R*_*c*_, we generate a probability matrix *R*_*p*_ to represent the preference probability between pairwise alternatives. The rule is: (i) *R*_*p*_(*A*_*i*_ ≻ *A*_*j*_) = 1 if *R*_*c*_(*i*, *j*) > 0.5; (ii) *R*_*p*_(*A*_*i*_ ≻ *A*_*j*_) = 0 if *R*_*c*_(*i*, *j*) ≤ 0.5. Hence,

$$R_{p}=\begin{array}{cc} & \begin{array}{cccc} AAU & Rg & Go8 & C9 \end{array}\\ \begin{array}{c} AAU\\ Rg\\ Go8\\ C9 \end{array} & \left(\begin{array}{cccc} 0 & 1 & 1 & 1\\ 0 & 0 & 1 & 1\\ 0 & 0 & 0 & 1\\ 0 & 0 & 0 & 0 \end{array}\right) \end{array}$$ (9)

Third, convert the probability matrix *R*_*p*_ to triangular matrix of probability $R_{p}^{T}$ using the triangularization method [23]. In particular, in the example the triangular matrix $R_{p}^{T}$ has the same form of *R*_*p*_, namely

$$R_{p}^{T}=\begin{array}{cc} & \begin{array}{cccc} AAU & Rg & Go8 & C9 \end{array}\\ \begin{array}{c} AAU\\ Rg\\ Go8\\ C9 \end{array} & \left(\begin{array}{cccc} 0 & 1 & 1 & 1\\ 0 & 0 & 1 & 1\\ 0 & 0 & 0 & 1\\ 0 & 0 & 0 & 0 \end{array}\right) \end{array}$$ (10)

According to $R_{p}^{T}$, the ranking of university alliances is obtained:

AAU≻Rg≻Go8≻C9 (11)

which means that AAU has the best scientific research ability, C9 has the worst performance, Rg and Go8 are located in the middle. The ranking is just a qualitative result. Based on the D-AHP approach, the quantitative priority weight of each university alliance can be obtained next.

Fourth, calculate the priority weights of university alliances. A triangulated crisp matrix $R_{c}^{T}$ is derived by integrating the crisp matrix *R*_*c*_ and triangular matrix $R_{p}^{T}$:

$$R_{c}^{T}=\begin{array}{cc} & \begin{array}{cccc} AAU & \;Rg & \;Go8 & \;\;C9 \end{array}\\ \begin{array}{c} AAU\\ Rg\\ Go8\\ C9 \end{array} & \left(\begin{array}{cccc} 0.5000 & 0.5805 & 0.6405 & 0.7644\\ 0.4195 & 0.5000 & 0.5868 & 0.7087\\ 0.3595 & 0.4132 & 0.5000 & 0.6260\\ 0.2356 & 0.2913 & 0.3740 & 0.5000 \end{array}\right) \end{array}$$ (12)

In matrix $R_{c}^{T}$, the elements above and alongside the main diagonal (namely 0.5805, 0.5868, and 0.6260) indicate the weight relationship of university alliances. We have

$$\left\{\begin{array}{c} \lambda (w_{AAU}-w_{Rg})=0.5805-0.5\\ \lambda (w_{Rg}-w_{Go8})=0.5868-0.5\\ \lambda (w_{Go8}-w_{C9})=0.6260-0.5\\ w_{AAU}+w_{Rg}+w_{Go8}+w_{C9}=1\\ \lambda >0,\\ w_{AAU},w_{Rg},w_{Go8},w_{C9}\geq 0 \end{array}\right.$$ (13)

**Fig 4:**
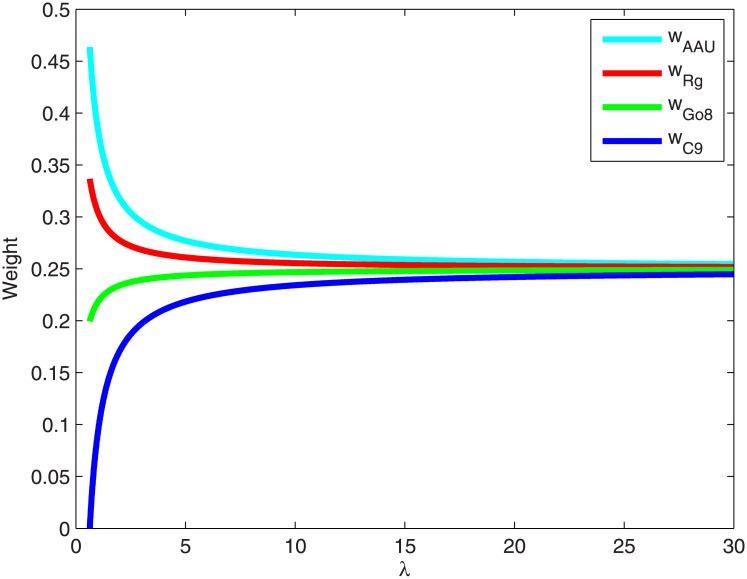
The priority weight of each university alliance with the change of *λ*.

By solving the above equations, we have

$$\left\{\begin{array}{c} w_{AAU}=1/4+0.135/\lambda \\ w_{Rg}=1/4+0.0548/\lambda \\ w_{Go8}=1/4-0.032/\lambda \\ w_{C9}=1/4-0.158/\lambda \\ \lambda \in [0.632,+\infty ] \end{array}\right.$$ (14)

where parameter *λ* expresses the credibility of information. If the comparison information is provided by an authoritative expert, *λ* takes a smaller value. If the comparison information comes from an expert whose judgment is with low belief, *λ* takes a higher value. The decline of *λ* means the drop of expert’s cognitive ability to slight difference. As a result, the weights of proposals are closing to each others. Fig 4 shows the priority weight of each university alliance with the change of *λ*.

With respect to the selection of *λ*, in [23] the authors proposed a scheme to determine the value of *λ*:

$$\lambda =\left\{\begin{array}{cc} \left\lceil \underline{\lambda }\right\rceil , & \text{The}\;\text{information}\;\text{is}\;\text{with}\;\text{high}\;\text{credibility}\\ \;\;n, & \text{The}\;\text{information}\;\text{is}\;\text{with}\;\text{medium}\;\text{credibility}\\ n^{2}/2, & \text{The}\;\text{information}\;\text{is}\;\text{with}\;\text{low}\;\text{credibility} \end{array}\right.$$ (15)

where $\underline{\lambda }$ represents lower bound of *λ*, $\left\lceil \underline{\lambda }\right\rceil =\text{min}\left\{k\in \mathbb{Z}|k⩾\underline{\lambda }\right\}$. And *n* is the number of alternatives.

In the study, we do not develop new scheme to determine the value of *λ*, but just simply use the scheme presented in [23]. According to such scheme, in this study we have: (i) *λ* = 1 if the information is with high credibility; (ii) *λ* = 4 if the information is with medium credibility; (iii) *λ* = 8 if the information is with low credibility. Therefore, the weights associated with different information credibility can be obtained, as shown in Table 9.

**Table 9:** Weights and ranking of university alliances under different credibility of information.

Universities	Weights (under different credibility of information)				Ranking
	High	Medium	Low	Interval	
AAU	0.385	0.284	0.267	(0.25, 0.464]	1
Rg	0.305	0.264	0.257	(0.25, 0.337]	2
Go8	0.218	0.242	0.246	[0.199, 0.25)	3
C9	0.092	0.210	0.230	[0, 0.25)	4

For the sake of comparison, we normalize all weights in interval [0, 100] by dividing the maximum one, and the results are shown in Table 10. From Table 10, we find that AAU always has the highest scores which indicate that it has the best scientific research ability. By contrast, C9’s scores of scientific research ability are always the lowest, especially it is 23.9 under high information credibility. Therefore, the results show that C9 falls behind the other university alliances in the aspect of scientific research ability, and the overall ranking is *AAU* ≻ *Rg* ≻ *Go*8 ≻ *C*9.

**Table 10:** The score of scientific research ability for different university alliances.

University alliance	Score (under different credibility of information)		
	High	Medium	Low
AAU	100	100	100
Rg	79.1	92.9	96.2
Go8	56.6	85.3	92.2
C9	23.9	74.2	86.3

## 6 Sensitivity analysis

In the section, several different settings of factors’ weights have been investigated to study the impact of change of weights on the evaluation result. It is noted that we only compare the results in the situation of high information credibility assumed by the D-AHP approach.

### 6.1 Reducing the weight of Quantity

Some experts may argue that the weight of Quantity which is 0.2 as shown in Fig 3 is too high. Now we reduce it to 0.1 and assign the remainder 0.1 to Quality or Influence, respectively. Assume that Case 1 means Weight(Quantity, Quality, Influence) = (0.2, 0.4, 0.4), Case 2 means Weight(Quantity, Quality, Influence) = (0.1, 0.5, 0.4), Case 3 means Weight(Quantity, Quality, Influence) = (0.1, 0.4, 0.5). The new results are given in Table 11.

**Table 11:** The scores of university alliances under different weight setting among Quantity, Quality, and Influence.

	Case 1	Case 2	Case 3
AAU	100	100	100
Rg	79.1	83.7	85.0
Go8	56.6	64.2	66.3
C9	23.9	26.1	22.9

From Table 11, it is found that reducing the weight of Quantity can obviously increase the sores of Rg and Go8 either in Case 2 or in Case 3, however it slightly increases the score of C9 in Case 2 and decreases the score of C9 in Case 3. These results imply that AAU has a distinct advantage in Quantity. But if the importance of Quantity is reduced, Rg and Go8 could narrow the gap with AAU. However, the means does not always work for C9, it must invest more effort on enhancing its Influence in the future.

### 6.2 Reducing the weight of %DC and increasing the weight of CI

In the case, we reduce the weight of %DC and increase the weight of CI, keeping the weight of TC unchanged. The new results are given in Table 12. In that Table, Case 1 indicates Weight(TC, CI, %DC) = (0.15, 0.50, 0.35), Case 2 indicates Weight(TC, CI, %DC) = (0.15, 0.60, 0.25), Case 3 indicates Weight(TC, CI, %DC) = (0.15, 0.70, 0.15), Case 4 indicates Weight(TC, CI, %DC) = (0.15, 0.80, 0.05), respectively.

**Table 12:** The scores of university alliances under different weight setting among TC, CI, and %DC.

	Case 1	Case 2	Case 3	Case 4
AAU	100	100	100	100
Rg	79.1	79.0	78.9	78.8
Go8	56.6	56.1	55.7	55.2
C9	23.9	22.7	21.5	20.4

According to Table 12, it is found that, as the decreasing the weight of %DC and increasing the weight of CI, the gap between Rg and AAU slightly ascends, so as the gap between Go8 and AAU, however the gap between C9 and AAU rises apparently. Therefore, the gap between C9 and AAU in the aspect of CI is more obvious than that in the aspect of %DC. So, in order to enhance C9 in the aspect of Quality more quickly, the decision maker should pay more attention on promoting the citation impact of papers.

## 7 Comparison and discussion

In this section, the results obtained by using the D-AHP approach are compared with that obtained by using other methods, to verify the effectiveness and reasonability of this study. What’s more, the performance of university alliances on each factor is assessed respectively to explore the measures of promoting the scientific research ability of university alliances.

Firstly, Table 13 gives the comparison of university alliances’ scientific research ability by using different methods including the D-AHP, conventional AHP [9] and TOPSIS [10]. Herein, the results of D-AHP are associated with the case of high information credibility. And in AHP method, the pairwise comparison matrix is generated through converting the D matrix in Eq (8) by using transformation equation *a*_*ij*_ = 3^2(2*r*_*ij*_−1)^ [43], then the classical eigenvector method [44] is employed to calculate the weight of each alliance, finally all weights are normalized in [0, 100] by dividing the maximum one. The TOPSIS is also a very popular MCDM method, the process of applying TOPSIS to MCDM problems can be clearly found in [45]. In this paper, the used TOPSIS is classical crisp-valued TOPSIS method since the collected data given in Table 6 are crisp values. From Table 13, it is found that these methods generate the same ranking *AAU* ≻ *Rg* ≻ *Go*8 ≻ *C*9, which verifies the reasonability of the results obtained by using the D-AHP approach. In addition, by investigating the concrete values in Table 13, we find that the score generated by the D-AHP and AHP are similar, but the score 2.2 coming from the TOPSIS is a little weird. If setting the score of AAU’s performance is 100, based on the TOPSIS, the score of C9 is only 2.2, it is a little counterintuitive. Therefore, the D-AHP and AHP is more effective in the application.

**Table 13:** Comparison of university alliances’ scientific research ability by using different methods.

	D-AHP	AHP (Eigenvector Method)	TOPSIS
AAU	100	100	100
Rg	79.1	74.3	51.5
Go8	56.6	52.5	39.5
C9	23.9	30.4	2.2

**Fig 5:**
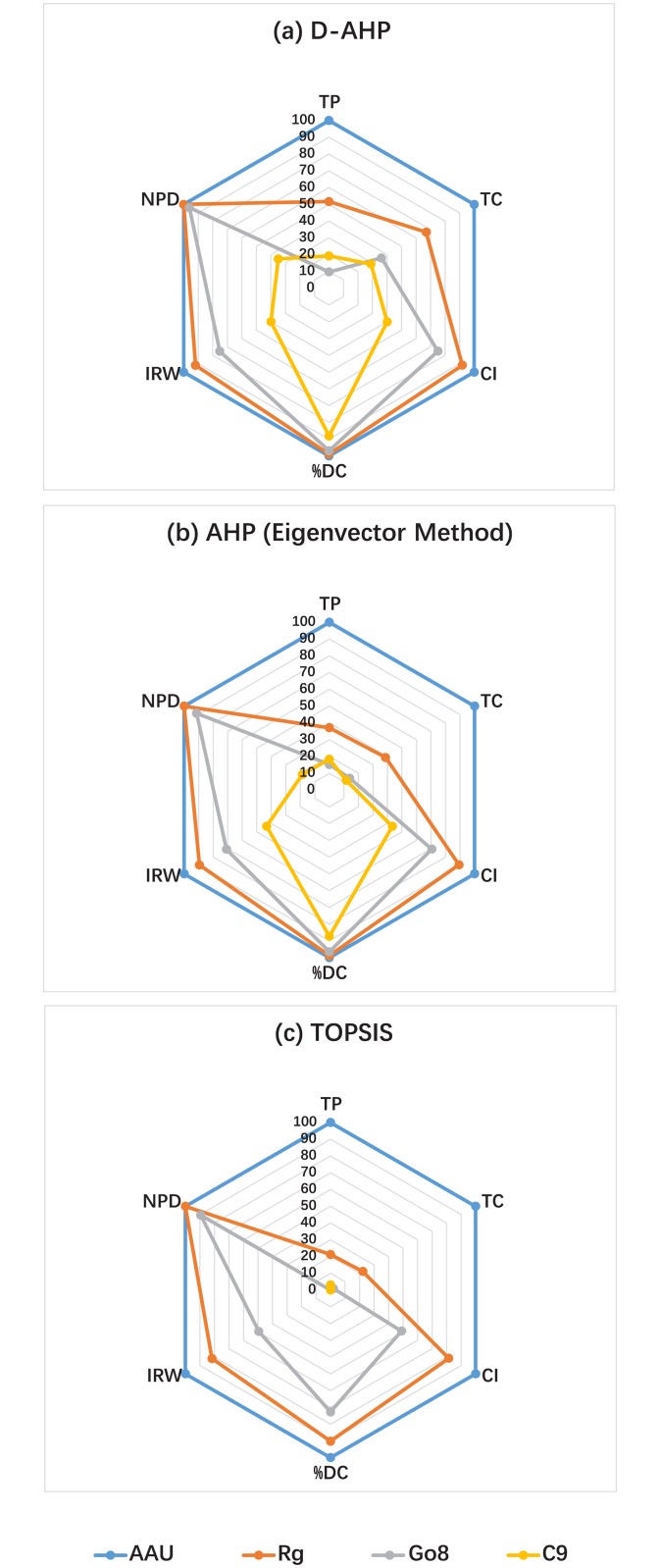
University alliances’ scores calculated by different methods while considering each assessment factor respectively.

Secondly, let us investigate the scores of university alliances while considering each assessment factor respectively. Tables 14, 15 and 16 are associated with the cases of D-AHP, AHP with eigenvector method, and TOPSIS, respectively. These results are graphically illustrated in Fig 5. In Figs 5(a) and 5(b), associated with the use of D-AHP and AHP respectively, AAU gets 100 score on every assessment factor, and C9 always performs the worst on all factors except TP where Go8 does the worst, Rg and Go8 are in the middle in most cases. On the other hand, by especially considering C9, it is very close to other university alliances in the aspect of %DC, but falls behind very much in other aspects. The score rankings of C9 on these factors are TP < TC < NPD < IRW < CI < %DC in the case of D-AHP and TC < TP < NPD < IRW < CI < %DC in the case of AHP. The two rankings are basically consistent. These rankings provide valuable reference in reducing the gap between C9 and world first-class university alliances. For China’s policy makers:

- The quality of publications should be more and more emphasized through a variety of ways, because the score on TC is very low which means that these publications can not get much attention. The reasons are complicated. For example, domestic researchers may pay too much interest on some outdated research topics or fields, facing that the policy makers must reduce the funding support on related fields so as to force researches to transfer to new research directions.
- The quantity of publications can give less attention. Although the score of C9 on TP is very low, but C9 just consists of nine universities. Compared with Go8 which has 8 affiliated universities, the total publications of C9 already has a little advantage. AAU and Rg get high scores because they are composed by more universities. Therefore, C9 just needs to keep current increasing rate of publications.
- The coordinated and balanced development of multiple disciplines must be encouraged with much more strength. According to the rankings, for C9 the NPD score is the third-lowest. From Table 5, C9 just owns three preponderant disciplines which are “Agricultural Sciences”, “Mathematics” and “Plant & Animal Science”. On one hand, the number of preponderant disciplines is few. On the other hand, these preponderant disciplines are all traditional disciplines. Therefore, the policy makers must pay more attention on the development of emerging disciplines by various means to implement the coordinated and balanced development of multiple disciplines.

**Table 14:** University alliances’ scores calculated by using the D-AHP approach while considering each assessment factor respectively.

	TP	TC	CI	%DC	IRW	NPD
AAU	100	100	100	100	100	100
Rg	51.7	66.9	91.8	98.9	91.9	100
Go8	9.7	36.1	75	96.9	75.2	96.1
C9	19.3	28.9	40.1	88.1	39.9	34.9

**Table 15:** University alliances’ scores calculated by using the AHP with eigenvector method while considering each assessment factor respectively.

	TP	TC	CI	%DC	IRW	NPD
AAU	100	100	100	100	100	100
Rg	37.1	38.7	89.3	98.7	89.4	100
Go8	15.2	14.0	70.5	96.5	70.8	91.4
C9	18.4	11.6	43.4	87.3	43.2	18.6

**Table 16:** University alliances’ scores calculated by using the TOPSIS method while considering each assessment factor respectively.

	TP	TC	CI	%DC	IRW	NPD
AAU	100	100	100	100	100	100
Rg	21.3	22.2	81.4	90.2	81.7	100
Go8	0	2.0	48.9	72.7	49.5	89.5
C9	3.1	0	0	0	0	0

Correspondingly, according to Fig 5(c) associated with the case of TOPSIS, although the ranking of university alliances on each factor is the same with the cases of D-AHP and AHP, the score of Go8 on TP and the scores of C9 on all factors except TP are all 0s. It is obviously unreasonable. Moreover, based on these scores, the performance of C9 on factors TC, CI, %DC, IRW, and NPD, can not be differentiated.

Through the above two aspects of comparisons, the effectiveness and reasonability of using the D-AHP in the study are shown. By contrast, the conventional TOPSIS is not appropriate for this work since it generates many counterintuitive results. The AHP method could produce reasonable results, but the collected data given in Table 6 is not in the form of pairwise comparison matrix, the AHP method can not be directly used in this application. Therefore, the D-AHP approach is more suitable than the AHP for this study.

## 8 Conclusion

In this paper, the issue of university scientific research ability evaluation has been studied. Four university alliances including AAU from North America, Rg from UK, Go8 from Australia, and C9 from China, have been chosen to illustrate the evaluation process. Data coming from InCites have been collected first. Then, a hierarchical structure has been built for the evaluation task. Within the study, a data-driven approach has been proposed to automatically construct the D matrix. After that, a new MCDM method called D-AHP model is utilized to evaluate and rank the scientific research ability of these university alliances. Next, a sensitivity analysis is conducted on the weights of factors and sub-factors within the established hierarchical structure of evaluation. Finally, the results obtained by using different methods are compared and discussed to verify the effectiveness and reasonability of this study, and some suggestions are given to promote China’s scientific research ability. The contribution of the work contains these aspects. At first, a new framework for the university scientific research ability evaluation is constructed, and it can be extended and enriched in other evaluation tasks of universities in the future. Secondly, a data-driven approach is proposed to automatically generate the D numbers preference relations, which is an originality for the research of D numbers. Thirdly, the latest data 2003–2013 are used to evaluate the scientific research ability of C9, which gives a fresh information on the research performance of C9. Fourthly, some suggestions to improve China’s scientific research ability, for example emphasizing the quality of publications and focusing on coordinated and balanced development of multiple disciplines, are given based on the analysis of concrete data. The limitation of the study is that the established assessment indicator structure is mainly based on universities’ performance on publications, which is not sufficient to comprehensively evaluate the performance of universities. The future research plan is to improve the assessment indicator structure to elevate its comprehensiveness and rationality.
